# Associations Between a Polymorphism in the Rat 5-HT_1A_ Receptor Gene Promoter Region (rs198585630) and Cognitive Alterations Induced by Microwave Exposure

**DOI:** 10.3389/fpubh.2022.802386

**Published:** 2022-02-17

**Authors:** Haijuan Li, Yu Gao, Yong Zou, Simo Qiao, Weijia Zhi, Lizhen Ma, Xinping Xu, Xuelong Zhao, Junhua Zhang, Lifeng Wang, Xiangjun Hu

**Affiliations:** ^1^Beijing Institute of Radiation Medicine, Beijing, China; ^2^Department of Urology, Chinese People's Liberation Army General Hospital, Beijing, China; ^3^Beijing Institute of Pharmacology and Toxicology, Beijing, China; ^4^Department of Emergency, Jingxi Medical District of Chinese People's Liberation Army General Hospital, Beijing, China

**Keywords:** microwave, 5-HT_1A_ receptor, SNP, cognition, association

## Abstract

The nervous system is a sensitive target of electromagnetic radiation (EMR). Chronic microwave exposure can induce cognitive deficits, and 5-HT system is involved in this effect. Genetic polymorphisms lead to individual differences. In this study, we evaluated whether the single-nucleotide polymorphism (SNP) rs198585630 of 5-HT_1A_ receptor is associated with cognitive alterations in rats after microwave exposure with a frequency of 2.856 GHz and an average power density of 30 mW/cm^2^. Rats were exposed to microwaves for 6 min three times a week for up to 6 weeks. PC12 cells and 293T cells were exposed to microwaves for 5 min up to 3 times at 2 intervals of 5 min. Transcriptional activity of 5-HT_1A_ receptor promoter containing rs198585630 C/T allele was determined *in vitro*. Electroencephalograms (EEGs), spatial learning and memory, and mRNA and protein expression of 5-HT_1A_ receptor were evaluated *in vivo*. We demonstrated that transcriptional activity of 5-HT_1A_ receptor promoter containing rs198585630 C allele was higher than that of 5-HT_1A_ receptor promoter containing T allele. The transcriptional activity of 5-HT_1A_ receptor promoter was stimulated by 30 mW/cm^2^ microwave exposure, and rs198585630 C allele was more sensitive to microwave exposure, as it showed stronger transcriptional activation. Rats carrying rs198585630 C allele exhibited increased mRNA and protein expression of 5-HT_1A_ receptor and were more susceptible to 30 mW/cm^2^ microwave exposure, showing cognitive deficits and inhibition of brain electrical activity. These findings suggest SNP rs198585630 of the 5-HT_1A_ receptor is an important target for further research exploring the mechanisms of hypersensitivity to microwave exposure.

## Introduction

In recent years, with the development of modern technology, electromagnetic radiation (EMR) has increasingly become a public health concern. Therefore, increasing attention has been paid to the biological effects and mechanisms of EMR. Microwave is the electromagnetic wave with frequency between 300 MHz and 300 GHz and microwave with a frequency of 2.856 GHz was widely used in radar and other communication devices. The nervous system is one of the most sensitive target of microwave radiation. Experimental studies show that exposure to microwave may lead to headache, sleep disorder, changes in EEG and cognitive function (1–3). And the research on the effect of microwave radiation on cognitive function has lasted for decades, but it is still controversial and the associated mechanisms remain unclear.

The 5-hydroxytryptamine (5-HT) play an important role in the cognition, emotion and brain development. Some evidence has indicated 5-HT and its receptors regulate the cognitive functions, including learning and memory (4–7). The main mechanism underlying the modulatory effects of 5-HT is an alteration in 5-HT receptor density during memory formation and in amnesic states (8, 9). The 5-HT receptor family includes at least 7 classes (5-HT_1−7_ receptor) and 15 subtypes with different functional and transduction properties. The 5-HT_1A_ subtype is of particular interest since it plays an important regulatory role in the 5-HT system as one of the main mediators of the action of 5-HT and a potential target for enhancing cognition (8, 10–12). The 5-HT_1A_ receptors can influence the activity of cholinergic, glutamatergic and GABAergic neurons in the cerebral cortex and hippocampus to affect the declarative and non-declarative memory. And the 5-HT_1A_ receptors regulate several transduction mechanisms in the memory formation. Perez-Garcia G et al. speculated that the mRNA expression of 5-HT_1A_ receptor in important brain regions may be used as a specific neuromarker of explicit memory and implicit memory (13). Elucidating the transcription and regulation of 5-HT_1A_ receptor will help to determine its mode of action in the nervous system. Our previous study indicated that long-term exposure to microwaves (2.856 GHz, average power densities of 10, 20 and 30 mW/cm^2^) can induce dose-dependent deficits in brain cognitive function. The activity of monoamine oxidase in the hippocampus decreased, resulting in the increase of 5-HT content and 5-HT_1A_ receptor expression which may mediate the disruption of spatial learning and memory caused by 30 mW/cm^2^ microwave exposure (14).

Genetic polymorphisms lead to individual differences. Single-nucleotide polymorphisms (SNPs) in the 5-HT_1A_ receptor gene promoter region regulate the expression of the 5-HT_1A_ receptor in human and animal models (15–17). Whether SNPs of the 5-HT_1A_ receptor gene play a role in susceptibility to the biological effects of EMR is still unclear.

In this study, we screened the SNP sites of rat 5-HT_1A_ receptor, and then we investigated the function and the association of the SNP site rs198585630 in the promoter region of rat 5-HT_1A_ receptor, which was detected in the screen results, with the cognitive deficit induced by microwave exposure.

## Materials and Methods

### Animals, Groups, and Genotyping of rs198585630

Male Wistar rats (*n* = 95) weighing 140–160 g were obtained from different laboratory animal centers (Beijing, China) and maintained at 22 ± 2°C under a 12-h light-dark cycle. Food and water were freely available. Genomic DNA was extracted from whole blood samples of the rats with a Quick Gene DNA Isolation Kit (Fujifilm, Japan). The extracted genomic DNA was then amplified by polymerase chain reaction (PCR) using the following primers: 5′-AGTGCCCCAGGATAGGTTAA-3′ (forward) and 5′-CACGTCGGAGATGCTAGTAA-3′ (reverse). PCR was performed as follows: initial denaturation for 5 min at 94°C; 35 cycles of 50 s at 94°C, 50 s at 56°C and 1 min at 72°C; and a final extension for 10 min at 72°C, terminating the PCR. The amplified products (1,192 bp) were identified by electrophoresis on a 1% agarose gel. The SNP site rs198585630 was detected in the promoter region of rat 5-HT_1A_ receptor. Then, rs198585630 genotype was determined by direct sequencing.

The rats were grouped into 3 genotype groups according to the sequencing results: rs198585630 TT, rs198585630 TC and rs198585630 CC groups. Rats in the same genotype group were then randomly divided into the control (C) group and microwave exposure (E) group. There were 16 rats in TT-C group, 16 rats in TC-C group, 17 rats in TT-E group, 17 rats in TC-E group, 14 rats in CC-C group and 15 rats in CC-E group. This study was approved by the Animal Care and Use Committee of Beijing Institute of Radiation Medicine.

### Cell Culture

PC12 cells and 293T cells were used to perform the *in vitro* functional studies. 293T cells were cultured in DMEM (Gibco, USA) supplemented with 10% fetal bovine serum (FBS; Gibco, USA) and penicillin (100 U/ml)/streptomycin (0.1 mg/ml) (Thermo, USA). PC12 cells were cultured in DMEM supplemented with 10% FBS, 5% horse serum (Gibco, USA) and penicillin (100 U/ml)/streptomycin (0.1 mg/ml). PC12 cells were plated in culture plates that precoated with 0.1 mg/ml poly-D-lysine (Invitrogen, USA). All cells were kept at 37°C in a humidified atmosphere of 95% air and 5% CO_2_.

### Microwave Exposure

Microwaves with a frequency of 2.856 GHz and an average power density of 30 mW/cm^2^ were generated by microwave exposure apparatus described in our previous study (18). In short, the microwave source is a klystron amplifier model JD 2000 (Vacuum Electronics Research Institute, Beijing, China). Microwave energy was transmitted by rectangular waveguide and A16-dB standard-gain horn antenna to an electromagnetic shield chamber. The diagonal of the antenna was 33 cm. The distance from the antenna to the top of the animal cage or cell culture dishes was 1.4 m. The average power density was measured using a waveguide antenna, the GX12M1CHP power meter (Guanghua Microelectronics Instruments, Hefei, China) and GX12M30A power heads.

Based on the previous studies, the dose of microwave radiation that can cause cognitive impairment was used (14). The rat container was round and made of Plexiglas with 20 houses. Each house could be adjusted so that the rats were comfortably placed and heads toward the antenna. The whole bodies of the rats were exposed to microwaves for 6 min three times a week for up to 6 weeks, and the average special absorption rate (SAR) of brain was calculated to be 17 W/kg. PC12 cells and 293T cells were cultured in the 6-wells plates and exposed to microwaves for 5 min up to 3 times at 2 intervals of 5 min, and the average SAR was 19W/kg. Rats and cells in the control group were processed parallel to those in the exposure groups, except for microwave radiation.

### Dual-Luciferase Reporter Assay

**Figure 1:**
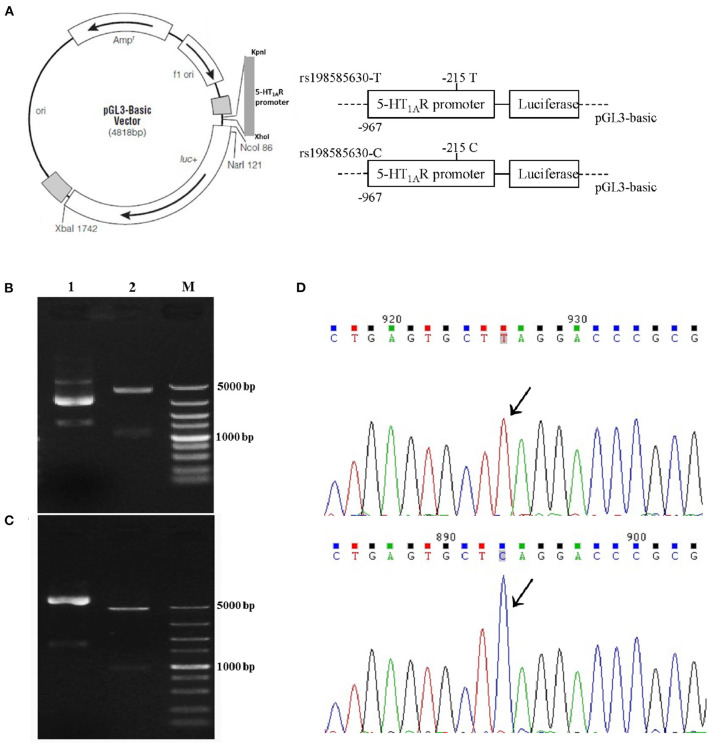
Recombinant reporter plasmids carrying the 5-HT_1A_ receptor rs198585630 T and C alleles. **(A)** The recombinant reporter plasmids. **(B,C)** Results of double digestion of the 5-HT_1A_ receptor promoter containing the−215 T allele **(B)** and C allele **(C)** in the pGL3-basic vector. Lane 1: Undigested fragment; lane 2: digested with KpnI and XhoI enzymes; M: DNA marker. **(D)** Sequencing of the 5-HT_1A_ receptor promoter containing the−215T allele and the C allele in the pGL3-basic vector.

Transcription of the rat 5-HT_1A_ receptor gene is initiated from a major site located between the −967 bp nucleotide and the initial ATG codon (19). We cloned 967 bp fragments including the SNP rs198585630, into the luciferase reporter plasmid pGL-3 Basic mRNA and protein (Promega, USA) to generate recombinant reporter plasmids carrying the SNP rs198585630 T allele and C allele separately (Figure 1A). The plasmids were verified by double digestion (Figures 1B,C) and sequencing (Figure 1D).

To determine the effect of the SNP rs198585630 on 5-HT_1A_ receptor promoter activity, the transcriptional activity of both versions of the promoter (the T allele and C allele) was examined in PC12 cells and 293T cells. Each of the three reporter plasmids (pGL3-Basic, pGL3-Basic-rs198585630-T and pGL3-Basic-rs198585630-C) was transiently transfected into cells using Lipofectamine 2000 (Invitrogen, USA). The pRL-TK vector was cotransfected (1:100) to normalize the transfection efficiency. At 6 h after transfection, the cells were exposed or sham-exposed to microwaves. Forty-two hours later, the cells were collected, and luciferase activity was measured using the Dual-Luciferase Reporter^®^ Assay System (Promega, USA) following the manufacturer's instructions.

### Electrophoretic Mobility Shift Assay

PC12 cells were harvested 1 h after microwave exposure or sham-exposure. After that, nucleoprotein was extracted from the cells using NE-PER^®^ nuclear and cytoplasmic extraction reagents 78833 (Pierce, USA). The concentration of the extracted nucleoprotein was quantified using a bicinchoninic acid (BCA) assay, and the nucleoprotein was stored at −80°C for subsequent studies.

*In silico* analysis by Gene-regulation (http://gene-regulation.com/) and MatInspector predicted that the most likely regulatory transcription factor that binds to the promoter containing rs198585630 is Nkx-2.5/Csx. The transcription factor binding site (TFBS) was gcctcTGAGtgct[t/c]aggac (the SNP rs198585630 is shown in square brackets). Probes (biotin-labeled or non-biotin-labeled) containing rs198585630 T/C allele were used for the EMSA. The double-stranded probes were generated by annealing a primer with the sequence 5′-GAGCCTCTGAGTGCT***T***AGGACCCGCGGGAG-3′ (for rs198585630 T allele) with its reverse complement primer and a primer with the sequence 5′-GAGCCTCTGAGTGCT***C***AGGACCCGCGGGAG-3′ (for rs198585630 C allele) with its reverse complement primer (the nucleotide corresponding to the SNP rs198585630 is shown in ***bold***). The CREB probes (5′-AGAGATTGCCTGACGTCAGAGAGCTAG-3′ and its reverse complement primer) were used as positive controls. The final concentrations of biotin-labeled and non-biotin-labeled deoxynucleotides were 20 fmol/μl and 4 pmol/μl, respectively.

The EMSA was performed according to the manufacturer′s instructions (LightShift^®^ Chemiluminescent EMSA Kit, 20148, Pierce, USA). Binding reactions were performed for 20 min at room temperature. Each binding system (50 μl) included 40 fmol biotin-labeled probes, 8 μg nucleoprotein from PC12 cells, 1× binding buffer, 1 μg poly(dI•dC) and 0.1 pmol MgCl_2_. In the competition assays, 200× non-biotin-labeled probes were preincubated with nucleoprotein for 20 min at room temperature before the biotin-labeled probes were added. After the binding assay, the reaction solutions were loaded onto a 4% non-denaturing polyacrylamide gel that had been prerun at 100 V for 1 h in 0.5× TBE buffer [44.5 mM Tris base, 44.5 mM boric acid, 1 mM EDTA (pH 8.0)], resolved on the gel at 100 V, and then blotted onto Ny^+^ nylon membranes at 380 mA for 40 min in 0.5× TBE buffer. The membrane was placed facing upward on a dry paper towel when the transfer was completed. The transferred DNA was crosslinked to the membrane at a distance of ~5 cm from a UV lamp equipped with 254 nm bulbs for 30 min. Then, the biotin-labeled DNA was detected by chemiluminescence as follows. The membrane was blocked for 15 min and then incubated in streptavidin-horseradish peroxidase conjugate/blocking buffer solution for 15 min with gentle shaking. After being washed 4 times for 5 min each in wash solution, the membrane was incubated in the substrate equilibration buffer for 5 min with gentle shaking, incubated in the substrate working solution for 5 min without shaking and then exposed to X-ray film (Kodak, Rochester, NY).

### Behavioral Test and Electroencephalogram Recording

#### Morris Water Maze Test

The MWM apparatus consisted of a black circular pool (150 cm in diameter), surrounded by light blue curtains and filled with clean water (23 ± 0.5°C). A movable platform (12 cm in diameter) was immersed 1.5 cm below the water surface in the center of the fourth quadrant of the pool and was maintained the same position throughout the test. Rat behavior in the MWM test was digitally recorded with a SLY-MW system (Beijing Sunny Instrument Co. Ltd., Beijing, China). After completion of the long-term exposure protocol, rats were trained to find the platform in five consecutive daily sessions. The average escape latency (AEL) was recorded and analyzed to assess the spatial learning ability of the rats. The platform was removed, and the probe test was performed on the 14th day after exposure. The percentage of time that the rats stayed in the target quadrant was recorded and analyzed to evaluate the long-term memory of the rats. The time schedule of the MWM test is shown in **Figure 3A**.

#### EEG Recording

The rats were lightly anesthetized, and EEGs were recorded with a BIOPAC MP-150 system (USA) from each rat before microwave exposure and then on the 14th day after exposure. And power spectral analyses of EEG were performed on spontaneous EEG segments.

### Measurement of 5-HT and 5-HIAA Levels in the Hippocampus

Rats were anesthetized on the 14th day after exposure. Then, the rats' brains were removed and the hippocampi were isolated immediately on ice. The hippocampi were homogenized in 5% perchloric acid on ice for 15 min and then centrifuged at 15,000 rpm for 20 min at 4°C, and the supernatant was stored at −20°C for later analysis. Subsequently, the 5-HT and 5-HIAA contents in the supernatants were measured by high-performance liquid chromatography with an electrochemical detector (HPLC–ECD). The HPLC system and the mobile phase were described in a previous study (14).

### Assessment of 5-HT_1A_ Receptor Expression

#### qRT-PCR Analysis of 5-HT_1A_ Receptor mRNA Levels

Total RNA was extracted from dissected hippocampal samples on the 14th day after exposure using TRIzol reagent (Invitrogen, USA). RNA levels were quantified using a Nanodrop 1000 spectrometer (Thermo Scientific, UK). Reverse transcription of mRNA was performed using a First-Strand cDNA Kit (AT311, TransGen, China) and a One-Step RT-PCR Kit (AQ131, TransGen, China) with SYBR Green (TransGen, China) according to the manufacturer's instructions. Amplification was conducted using a 7500 real-time PCR system (Applied Biosystems, USA). GAPDH, a housekeeping gene, was used as an internal control to standardize 5-HT_1A_ receptor mRNA expression. Relative expression (R) of the 5-HT_1A_ receptor gene was calculated using the 2^−ΔCT^ method (ΔCT = CT_(5−HT1AR)_ - CT_(GAPDH)_). The primers were designed using Primer Premier 5.0 software. The primer sequences were as follows: 5-HT_1A_ receptor forward, 5′-AGTGAGGCAGGGTGACGACG-3′, and reverse, 5′-CAGGACCAGAGCCACAATGAAA-3′ (265 bp); GAPDH forward, 5′-GATTTGGCCGTATCGGAC-3′, and reverse 5′-GAAGACGCCAGTAGACTC-3′ (278 bp).

#### Western Blot Analysis of 5-HT_1A_ Receptor Protein Levels

On the 14th day after exposure, 5-HT_1A_ receptor protein expressions in the hippocampi of rats in the microwave exposure and control group of the different genotypes were measured by western blotting. Western blotting was performed as previously reported (20), and an antibody against the 5-HT_1A_ receptor (Millipore, USA) was used at 1:400 dilution. Quantification of the protein band intensity was carried out by Quantity One imaging software (Bio-Rad Laboratories, USA). The results of 5-HT_1A_ receptor levels were normalized to GAPDH levels (1:5,000, Kangchen Bio-tech, China).

### Data Analysis

All data are expressed as means ± Standard Error of Mean (SEM) (x ± s). Statistical analysis was performed using SPSS16.0 software, The statistical method of repeated measurement analysis of variance (ANOVA) was used for the positioning navigation data in Morris water maze and EEG data, and ANOVA was used to compare the differences among groups of other data. On this basis, multiple comparisons were performed by the SNK analysis. The accepted level of significance for all tests was *P* < 0.05 and the significance symbols were different *in vivo* and *in vitro* experiments which were shown in the figure legends.

## Results

### Functional Analysis of rs198585630 and Its Association With Microwave-Induced Cell Alterations

#### rs198585630 Modulates Transcriptional Activity

**Figure 2:**
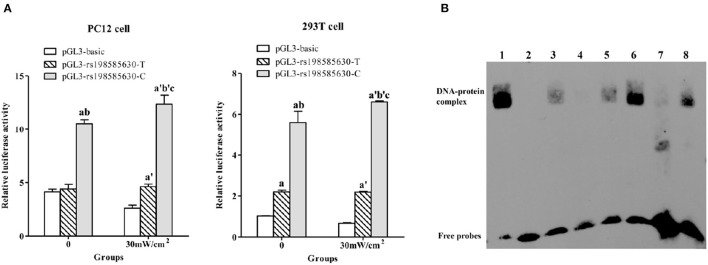
Functional analysis of rs198585630 and its association with the alterations induced by microwave exposure. **(A)** Relative luciferase activity driven by either the 5-HT_1A_ receptor promoter−215T or C allele-pGL3 plasmid in PC12 and 293T cells treated with or without 30 mW/cm^2^ microwave exposure. ^a^*P* < 0.05 vs. pGL3-basic-0, ^b^*P* < 0.05 vs. pGL3-T-0, ^c^*P* < 0.05 vs. pGL3-C-0, ^a^^′^*P* < 0.05 vs. pGL3-basic-30 mW/cm^2^, ^b^^′^*P* < 0.05 vs. pGL3-T-30 mW/cm^2^. **(B)** Analysis of 5-HT_1A_ receptor−215 T/C oligonucleotides incubated with nuclear extracts derived from PC12 cells by the EMSA. Lane 1: labeled CREB probe with nuclear extract from control cells; lane 2: labeled C probe without nuclear extract; lane 3: labeled C probe with nuclear extract from control cells; lane 4: labeled C probe and 200-fold unlabeled C probe with nuclear extract from control cells; lane 5: labeled C probe and 200-fold unlabeled T probe with nuclear extract from control cells; lane 6: labeled C probe with nuclear extract from 30 mW/cm^2^-exposed cells; lane 7: labeled T probe with nuclear extract from control cells; lane 8: labeled T probe with nuclear extract from 30 mW/cm^2^-exposed cells.

A dual-luciferase reporter assay was used to investigate the transcriptional activity of 5-HT_1A_ receptor fragments (967 bp) containing the −215 T/C allele under exposure and sham-exposure conditions. The trends observed in PC12 cells and 293T cells were basically consistent (Figure 2A). The luciferase activity of cells transfected with the pGL3-rs198585630-C plasmid was higher than that of cells transfected with the pGL3-rs198585630-T plasmid and pGL3-basic plasmid (*P* < 0.05). The results indicated that the transcriptional activity of the 5-HT_1A_ receptor fragments (967 bp) containing the −215 C allele was higher than that of 5-HT_1A_ receptor fragments containing the T allele.

After 30 mW/cm^2^ microwave exposure, the luciferase activity of cells transfected with the pGL3-rs198585630-C and pGL3-rs198585630-T plasmids was increased compared to that of cells transfected with the pGL3-basic plasmid (*P* < 0.05), and the activity of cells transfected with pGL3-rs198585630-C was increased compared to that of cells transfected with the pGL3-rs198585630-T plasmid (*P* < 0.05). Furthermore, following microwave exposure, the activity of cells transfected with the pGL3-rs198585630-C plasmid was increased compared with that of the control groups (*P* < 0.05). There were no significant changes between cells transfected with the pGL3-rs198585630-T plasmid or pGL3-basic plasmid after exposure and cells in the control groups. The results showed that the transcriptional activity of the fragments containing the −215 C allele was increased after 30 mW/cm^2^ microwave exposure, while this alteration was not observed for fragments containing the T allele.

#### rs198585630 Modulates the Recruitment of Transcription Factors to the Promoter

To determine whether rs198585630 regulates the binding of the transcription factor to the promoter and the change after microwave exposure, the EMSA was carried out. rs198585630 C/T variant site of the 5-HT_1A_ receptor was labeled with biotin to detect PC12 cell nuclear proteins in the EMSA. The results showed that the C and T alleles both bound to nuclear proteins, with higher levels of binding for the C allele than for the T allele. Furthermore, binding to the labeled C probes was competitively inhibited by the appropriate unlabeled C probes but not the unlabeled T probes. After 30 mW/cm^2^ microwave exposure, the amount of proteins bound to the C and T alleles was both increased, with a more significant effect observed for the C allele (Figure 2B). The results suggested that the binding of the transcription factor to the promoter containing the C allele was stronger than that to the promoter containing the T allele. Additionally, the alteration in binding after microwave exposure was more sensitive for promoters containing the C allele than those containing the T allele.

### Association of rs198585630 With Microwave-Induced Functional Brain Abnormalities

#### Spatial Learning and Memory

**Figure 3:**
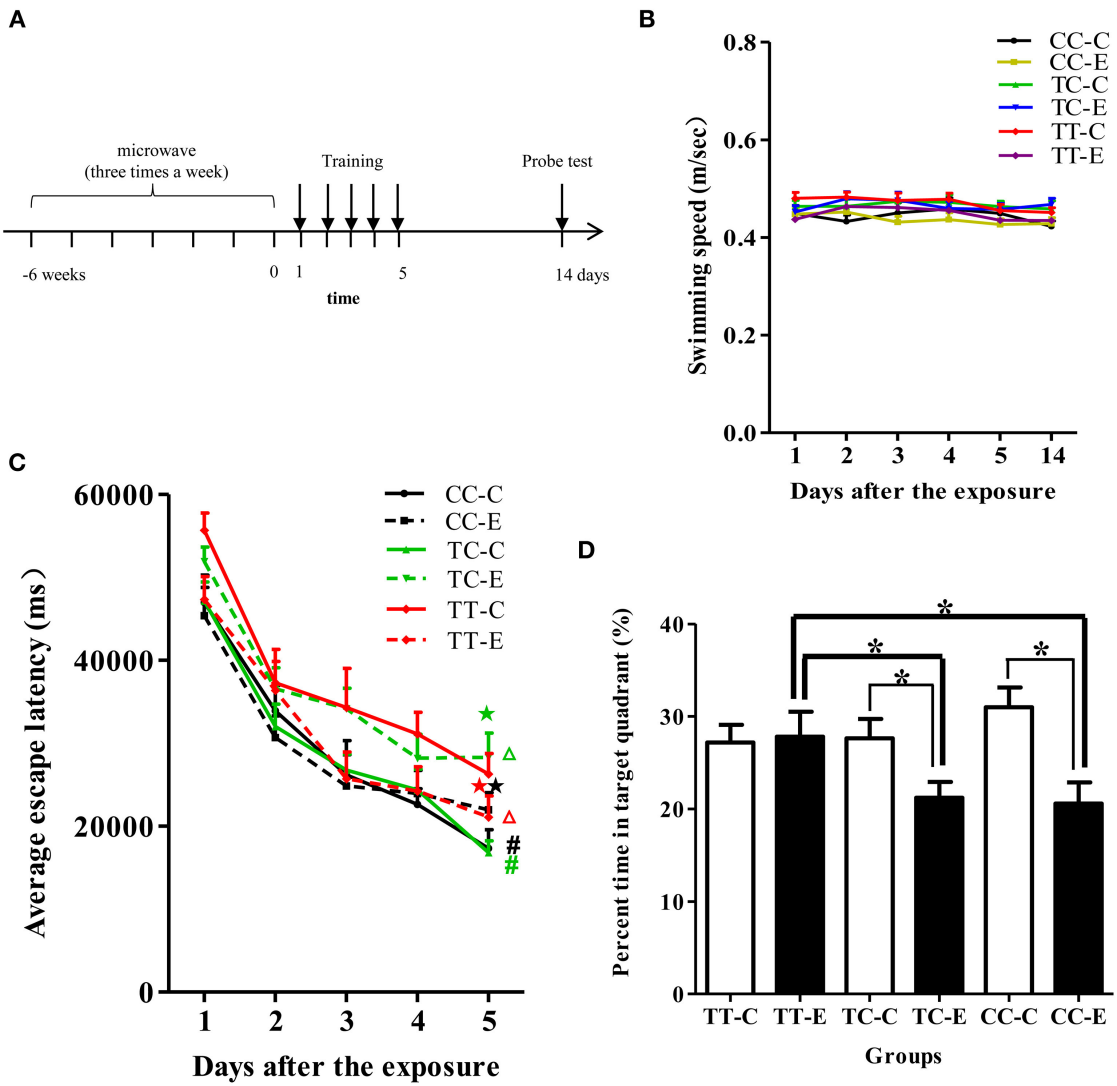
Performance of rats in the MWM test. **(A)** Time schedule of the experiments. **(B)** Swimming speed of the rats. **(C)** AELs in the navigation test. Statistical significance (repeated measures ANOVA); changes in the exposed TC genotype (TC-E) and exposed TT genotype (TT-E) groups: ^Δ^*P* < 0.05 vs. the control group of the same genotype; changes in the control TC genotype (TC-C) and control CC genotype (CC-C) groups: ^#^*P* < 0.05 vs. the control TT genotype (TT-C) group; ^⋆^*P* < 0.05 vs. control rats of the same genotype at the corresponding time point; **(D)** percentage of time spent in the target quadrant in the probe test. *P < 0.05.

According to repeated measures ANOVA, during the training session (the 1st day to the 5th day after the exposure), the control rats in the TT genotype group exhibited a longer AEL than those in the TC and CC genotype groups (*P* < 0.05), as shown in Figure 3C. At the end of the training sessions (5 days after exposure), the AEL of each group (including the exposure and control groups of the different genotypes) gradually decreased to a stable level, indicating that rats had acquired the ability to find the platform. The rats in the TC and CC genotype groups exposed to microwaves exhibited longer AELs than control rats of the same genotype (*P* < 0.05), while rats in the TT genotype group exhibited a decreased AEL (*P* < 0.05). Analysis of AELs suggested that in the absence of microwave exposure, rats of the TT genotype exhibited poorer spatial learning ability than those of the TC or CC genotype. Spatial learning ability was enhanced in rats of the TT genotype but decreased in rats of the TC and CC genotypes after 30 mW/cm^2^ microwave exposure. In the probe test (Figure 3D), the percentage of time spent in the target quadrant by rats in the TC and CC genotype groups was less than that by rats of the same genotype after exposure (*P* < 0.05). A lower percentage of time spent in the platform quadrant indicates poorer memory retention; thus, the results suggested that the spatial memory of rats of the TC and CC genotypes was decreased after exposure. Among the exposure groups, rats of the TT genotype spent a greater percentage of time in the target quadrant than rats of the TC and CC genotypes. Swimming speed did not differ between any of the groups (*P* > 0.05; Figure 3B).

#### EEG

**Figure 4:**
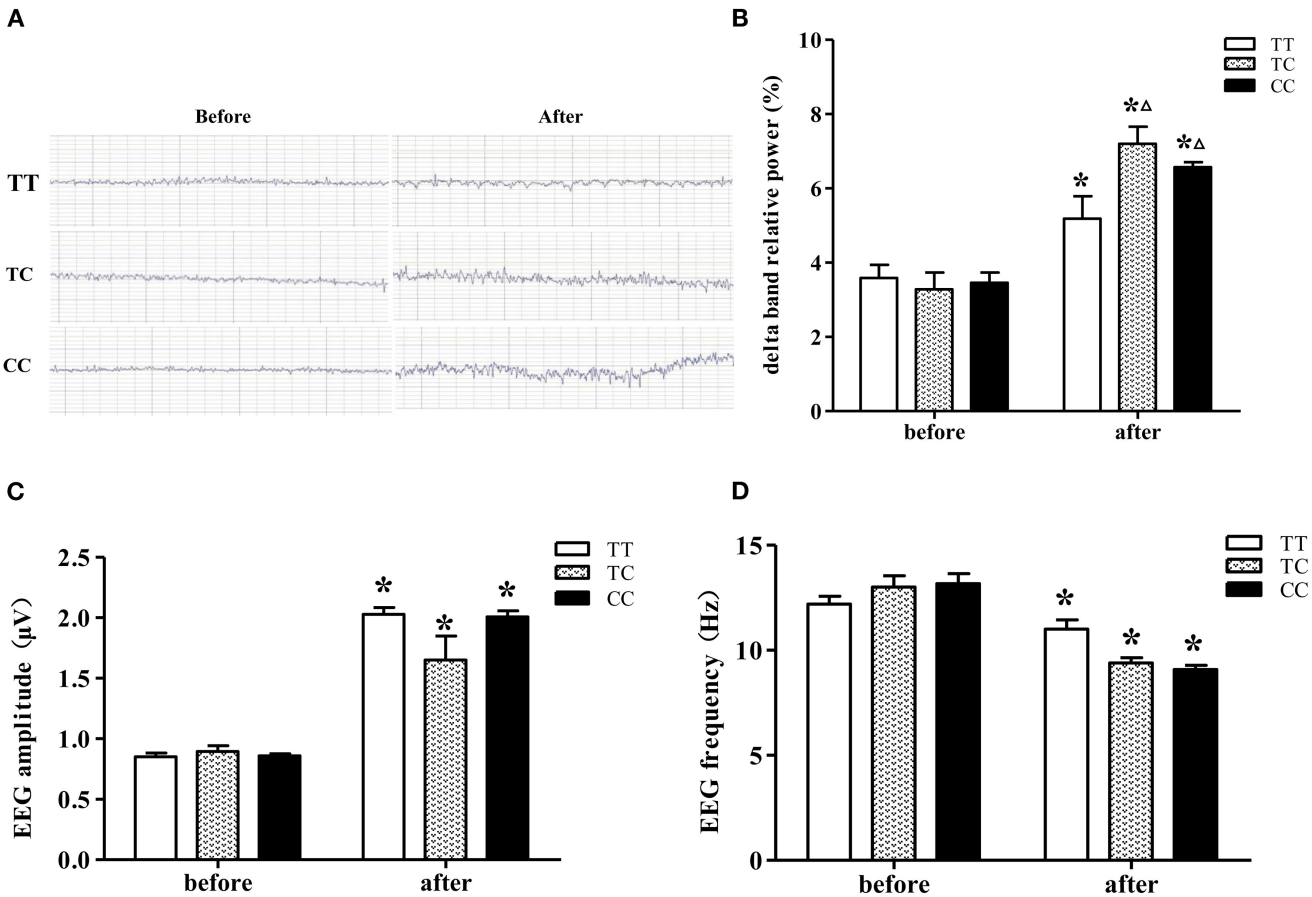
EEGs from rats on the 14th day after 30 mW/cm^2^ microwave exposure. **(A)** EEG. **(B)** Delta band relative power. **(C)** Amplitude. **(D)** Frequency. Statistical significance (repeated measures ANOVA); changes in the TC and CC genotype groups: ^Δ^*P* < 0.05 vs. the TT genotype group. *P < 0.05 vs. prior to exposure.

EEGs were recorded from rats (Figure 4A), and the delta band relative power, amplitude and frequency were assessed (Figures 4B–D). On the 14th day after exposure, delta band relative power and the amplitude were increased, the mean frequency was decreased in the rats of all three genotypes, which were exposed to microwaves compared to control rats of the same genotype (*P* < 0.05). In advance, the delta band relative power was significantly increased, and the increase was greater in rats of the TC and CC genotypes than those of the TT genotype. Among the exposure groups, the delta band relative power of rats in the TC and CC genotype groups was higher than that of rats in the TT genotype group, and there was no difference between the TC and CC genotype groups. The EEG results suggested that long-term microwave exposure inhibited brain electrical activity and that rats in TC and CC genotype groups were more sensitive to these changes than those in the TT genotype group.

### Association of rs198585630 With Microwave-Induced Alterations in the 5-HT System

#### 5-HT and 5-HIAA Levels in the Hippocampus

As shown in Table 1, there was a decreasing trend in 5-HIAA content and an increasing trend in 5-HT content in TT genotype group after exposure, although the differences were not significant (*P* > 0.05). The 5-HIAA/5-HT ratio was significantly lower in the TT genotype group exposed to microwaves than control rats in the TT genotype group (*P* < 0.05). There was no significant difference in 5-HIAA content, 5-HT content or the 5-HIAA/5-HT ratio between the exposure and control groups in rats of the TC and CC genotypes.

**Table 1:** 5-HT and 5-HIAA contents in rats' hippocampi (means ± SEM).

**Groups**	**Contents (pg/mg)**		
	**5-HIAA**	**5-HT**	**5-HIAA/5-HT**
TT-C	75.27 ± 6.96	99.11 ± 10.27	0.79 ± 0.07
TT-E	55.75 ± 7.18	118.00 ± 14.58	0.48 ± 0.04^*^
TC-C	59.68 ± 8.32	101.20 ± 11.16	0.58 ± 0.05
TC-E	69.12 ± 16.50	93.00 ± 14.94	0.68 ± 0.08
CC-C	58.01 ± 8.60	107.50 ± 17.43	0.56 ± 0.05
CC-E	67.98 ± 6.54	113.60 ± 8.51	0.60 ± 0.03

* *P < 0.05 vs. TT-C group*.

#### mRNA and Protein Expression of 5-HT_1A_ Receptor

**Figure 5:**
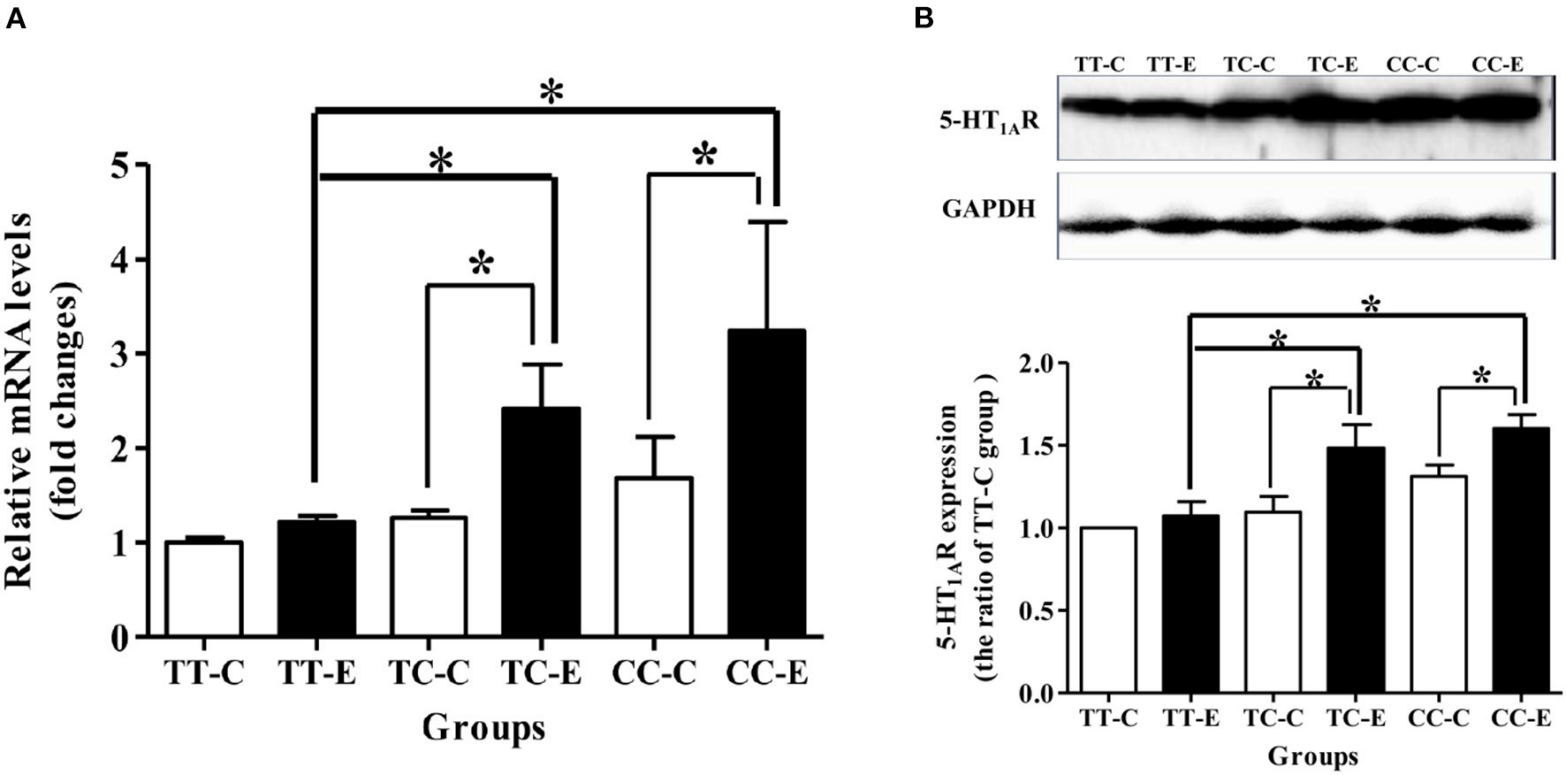
5-HT_1A_ receptor mRNA and protein levels in the rat hippocampus after 30 mW/cm^2^ microwave exposure. **(A)** mRNA level; **(B)** protein levels. *P < 0.05. The histogram panels show semiquantified protein level data. Each value is expressed as the ratio of the level in the indicated group to the level in the control TT genotype (TT-C) group. *P < 0.05.

As shown in Figures 5A,B, mRNA and protein expression of 5-HT_1A_ receptor was higher in rats of the TC and CC genotypes exposed to microwaves than control rats of the same genotype (*P* < 0.05). Rats in the TC and CC genotype groups exhibited higher mRNA and protein expression levels of 5-HT_1A_ receptor than the TT genotype group (*P* < 0.05).

## Discussion

The researches on the impact of microwave radiation on cognitive function include three aspects: promotion, inhibition and negative results (21). On the one hand, due to the complexity of microwave radiation parameters, including its frequency, power density, irradiation time and average absorption rate, the radiation parameters used in various studies are not identical, so it is difficult to obtain the comparable results. On the other hand, the cognitive function is complex and affected by many factors, such as a variety of neurotransmitters and their interactions. Gene polymorphism may cause changes in gene function, which is the genetic basis for the differences of biological traits among different individuals, such as susceptibility to complex diseases and drug treatment (22, 23). The difference of susceptibility to microwave radiation may be one of the important reasons for the differences in the studies, but the role of gene polymorphism in the susceptibility difference of biological effects of microwave radiation remains to be studied (24).

5-HT participates in the regulation of sensory, motor, learning and memory and other behavioral functions, and its role in cognitive behavior has attracted more and more attention, becoming a pharmacological and genetic target in the treatment of memory disorders (7, 9). The increase of 5-HT_1A_R density is related to the decline of cognitive function and hippocampal postsynaptic 5-HT_1A_R has a negative effect on explicit memory function (13, 25). The transcription of the rat 5-HT_1A_ receptor gene is mainly initiated by the sequence from −967 bp upstream to the ATG segment of the start codon (19). The site of rs198585630, which was identified in our previous study, is located in the promoter region of the 5-HT_1A_ receptor gene. No functional studies on this site have been reported yet. The premise for studying the functional significance of rs198585630 as a SNP in the promoter region is that regulation of 5-HT_1A_ receptor expression has physiological and pathological significance. Our previous study indicated that variations in 5-HT_1A_ receptor expression are involved in microwave exposure-induced cognitive deficits. rs198585630, which is located in the promoter region, has the potential to regulate the expression of 5-HT_1A_ receptor. Therefore, *in vivo* and *in vitro* experiments were conducted to study the function of rs198585630 and its association with individual differences in cognitive alterations after microwave exposure.

An *in vitro* study indicated that the transcriptional activity of 967bp 5-HT_1A_ receptor fragments containing the −215 C allele was higher than that of 967 bp 5-HT_1A_ receptor fragments containing the T allele. The SNP rs198585630 may be located at the TFBS, and the −215 T>C polymorphism affected transcription activity. Moreover, the promoters containing the C allele were more sensitive to alterations in transcriptional activity after microwave exposure than those containing the T allele.

Based on the *in vitro* study of rs198585630 function, *in vivo* studies were carried out to explore the associations between rs198585630 and alterations in 5-HT_1A_ receptor induced by microwave exposure. The mRNA and protein expression levels of 5-HT_1A_ receptor in the rats' hippocampi of the TC and CC genotypes increased significantly after exposure, which was basically consistent with the results of *in vitro* experiments. rs198585630 significantly affected the expression of 5-HT_1A_ receptor. rs198585630 C allele is related to higher expression of 5-HT_1A_ receptor and more sensitive to microwave exposure. Studies in rat and mouse models have provided evidence that performance in the MWM test is highly sensitive to changes in 5-HT_1A_ receptor function (13, 26, 27). Considering the important role of 5-HT_1A_ receptor in cognitive function, we tested the spatial learning and memory of rats of different genotypes and found that among rats in the control groups, the spatial learning ability of rats of the TT genotype was lower than that of rats of the TC and CC genotypes. The spatial learning ability of the TT genotype exposed to microwaves was enhanced compared to that of control of the same genotypes, whereas spatial memory was not significantly altered after microwave exposure. The spatial learning and memory of the TC and CC genotypes were both significantly reduced after exposure.

An increase in 5-HT_1A_ receptor density is related to a decline in cognitive function (28–30), and 5-HT_1A_ receptor expressed on hippocampal postsynaptic neurons has a negative effect on explicit memory function (11, 31). Stimulation of 5-HT_1A_ receptors usually leads to learning disabilities by interfering with memory coding mechanisms, while 5-HT_1A_ receptor antagonists promote certain types of memory by enhancing cholinergic and/or glutamatergic neurotransmission in hippocampus/cortex (13, 32, 33). Similarly, we observed that compared with rs198585630 T allele, rs198585630 C allele was related to higher transcriptional activity of the 5-HT_1A_ receptor promoter and increased mRNA and protein expression of 5-HT_1A_ receptor after microwave exposure. Rats carrying rs198585630 C allele were more susceptible to the spatial learning and memory deficits induced by microwave exposure.

The 5-HIAA/5-HT ratio reflects the relative metabolic rate of 5-HT, which can be used to evaluate serotonergic system activity (34–36). After microwave exposure, the 5-HIAA/5-HT ratio in rs198585630 TT rats was significantly decreased, reflecting decreased catabolism and increased excitability of 5-HT neurons. Considering the performance of the rats in the MWM test (increased spatial learning and no significantly change in memory), the decrease in the 5-HIAA/5-HT ratio may represent a mechanism compensating for decreased expression of 5-HT_1A_ receptor and leading to a return of the 5-HT system to the physiological range to maintain effective neurotransmission.

Alterations in EEGs can be seen in pathology and cognitive disorders, as these conditions involve cognitive deficits that are closely related to inhibition of EEG activity (37–39). The monoamine-acetylcholine balance hypothesis is a theory related to neurophysiological markers on EEG, and an increased delta frequency band reflects an increase in the effects of inhibitory monoamine receptor subtypes such as 5-HT_1A_ receptor (40). At a low dosage, a 5-HT_1A_ receptor agonist enhances EEG power in delta rage (41). The EEG results showed that after 30 mW/cm^2^ microwave exposure, the delta band relative powers of rats in the TC and CC genotype groups were higher than that of rats in the TT genotype group, which was consistent with the previously described changes in 5-HT_1A_ receptor expression after microwave irradiation.

## Conclusions

In summary, rs198585630 site in the rat 5-HT_1A_ receptor promoter region is a functional site that regulates the transcription of 5-HT_1A_ receptor. The transcriptional activity of the 5-HT_1A_ receptor promoter containing the −215 C allele is higher than that of the 5-HT_1A_ receptor promoter containing the T allele. The transcriptional activity of the 5-HT_1A_ receptor promoter was stimulated by 30 mW/cm^2^ microwave exposure, and the 5-HT_1A_ receptor rs198585630 C allele was more sensitive to microwave exposure, showing stronger transcriptional activation. Rats with rs198585630 C allele presented higher mRNA and protein expression of 5-HT_1A_ receptor and were more susceptible to 30 mW/cm^2^ microwave exposure, as indicated by cognitive deficits and brain electrical activity inhibition, than those with rs198585630 T allele. These findings suggest that the SNP rs198585630 of the 5-HT_1A_ receptor is an important target for further research exploring the mechanisms of hypersensitivity to microwave exposure.

## Data Availability Statement

The original contributions presented in the study are included in the article/supplementary material, further inquiries can be directed to the corresponding authors.

## Ethics Statement

The animal study was reviewed and approved by Institutional Animal Care and Use Committee of Beijing Institute of Radiation Medicine.

## Author Contributions

LW and XH: conceptualization and writing—review and editing. LW and WZ: funding acquisition and resources. HL: methodology. YZ: software. HL and YG: validation and data curation. LM and WZ: formal analysis. XZ: microwave exposure. XX: EEG. SQ: investigation. HL, YG, LM, and JZ: writing—original draft preparation. LM: visualization. WZ: supervision. All authors have read and agreed to the published version of the manuscript.

## Funding

This research was funded by grants of Logistics Research Program (China) (No. AWS17J006) and National Natural Science Foundation of China (No. 31800701).

## Conflict of Interest

The authors declare that the research was conducted in the absence of any commercial or financial relationships that could be construed as a potential conflict of interest.

## Publisher's Note

All claims expressed in this article are solely those of the authors and do not necessarily represent those of their affiliated organizations, or those of the publisher, the editors and the reviewers. Any product that may be evaluated in this article, or claim that may be made by its manufacturer, is not guaranteed or endorsed by the publisher.
